# The features of AECOPD with carbon dioxide retention

**DOI:** 10.1186/s12890-018-0691-8

**Published:** 2018-07-31

**Authors:** Xia Wei, Nan Yu, Qi Ding, Jingting Ren, Jiuyun Mi, Lu Bai, Jianying Li, Min Qi, Youmin Guo

**Affiliations:** 1 Department of Radiology, Xi’an Jiaotong University Medical College First Affiliated Hospital, Xi’an, China; 2 Department of Respiratory Medicine, The Ninth Hospital of Xi’an Affiliated Hospital of Xi’an Jiaotong University, Xi’an, China; 3 Department of Radiology, The Affiliated Hospital of Shaanxi University of Traditional Chinese Medicine, Xianyang, Shaanxi China; 4 Department of Respiratory Medicine, Central Hospital of Xi’an Affiliated Hospital of Xi’an Jiaotong University, Xi’an, Shaanxi China; 5 Department of Radiology, Shaanxi Provincial People’s Hospital, Xi’an, China

**Keywords:** Acute exacerbation, Chronic obstructive pulmonary disease, Pulmonary function test, Emphysema index, Carbon dioxide retention

## Abstract

**Background:**

Chronic obstructive pulmonary disease (COPD) with carbon dioxide retention is associated with a worsening clinical condition and the beginning of pulmonary ventilation decompensation. This study aimed to identify the factors associated with carbon dioxide retention.

**Methods:**

This was a retrospective study of consecutive patients with COPD (meeting the Global Initiative for Chronic Obstructive Lung Disease diagnostic criteria) hospitalized at The Ninth Hospital of Xi’an Affiliated Hospital of Xi’an Jiaotong University between October 2014 and September 2017. The baseline demographic, clinical, laboratory, pulmonary function, and imaging data were compared between the 86 cases with carbon dioxide retention and the 144 cases without carbon dioxide retention.

**Results:**

Compared with the non-carbon dioxide retention group, the group with carbon dioxide retention had a higher number of hospitalizations in the previous 12 months (*p* = 0.013), higher modified Medical Research Council (mMRC) dyspnea scores (*p* = 0.034), lower arterial oxygen pressure (*p* = 0.018), worse pulmonary function (forced expiratory volume in one second/forced vital capacity [FEV_1_/FVC; *p* < 0.001], FEV_1_%pred [p < 0.001], Z5%pred [*p* = 0.004], R5%pred [*p* = 0.008], R5-R20 [*p* = 0.009], X5 [*p* = 0.022], and Ax [*p* = 0.011]), more severe lung damage (such as increased lung volume [p = 0.011], more emphysema range [*p* = 0.007], and lower mean lung density [*p* = 0.043]). FEV_1_ < 1 L (odds ratio [OR] = 4.011, 95% confidence interval [CI]: 2.216–7.262) and emphysema index (EI) > 20% (OR = 1.926, 95% CI: 1.080–3.432) were independently associated with carbon dioxide retention in COPD.

**Conclusion:**

Compared with the non-carbon dioxide retention group, the group with carbon dioxide retention had different clinical, pulmonary function, and imaging features. FEV_1_ < 1 L and EI > 20% were independently associated with carbon dioxide retention in AECOPD.

**Trial registration:**

ChiCTR-OCH-14004904. Registered 25 June 2014.

**Electronic supplementary material:**

The online version of this article (10.1186/s12890-018-0691-8) contains supplementary material, which is available to authorized users.

## Background

Chronic obstructive pulmonary disease (COPD) is the fourth leading cause of death worldwide and is expected to be the third leading cause of death by 2020 [1]. COPD is also a major chronic disease that produces a large economic and social burden worldwide [2]. In China, COPD is a major contributor to the overall morbidity and mortality burden owing to the relatively high prevalence of smoking and rising environmental pollution [3, 4]. There are limited treatments available for the effective prevention of COPD progression. Respiratory failure secondary to AECOPD can lead to disease progression. Therefore, distinguishing patients with a risk of carbon dioxide retention is of clinical importance in the management of acute exacerbation of chronic obstructive pulmonary disease (AECOPD).

Since COPD is a heterogeneous disease, it is difficult for a single indicator to reflect all features of the disease. Pulmonary function indicators, especially FEV_1_ (forced expiratory volume in 1 s), are recognized as important because they can reflect one of the key characteristics of COPD - airflow limitation, but according to the Global Initiative for Chronic Obstructive Lung Disease (GOLD) update in 2017 [5], FEV_1_ was used for grading of disease severity but was not a variable used to guide treatment. Other well accepted indicators include 64-detector computed tomography (CT) parameters such as bronchial wall thickness and emphysema index (EI), which reflect COPD pathological changes [6, 7], airway wall thickening, and airway remodeling response. Emphysema index refers to the proportion of low-density areas less than − 950 HU occupying the lung volume. An increase in the emphysema index reflects an increase in the extent of parenchymal destruction of the lungs. Yamasawa [8] reported that CT could be used as a non-invasive tool to predict aerobic capacity in COPD.

Another indicator that holds promise for assessing the severity of COPD is carbon dioxide retention. Carbon dioxide retention indicates the exhaustion of lung reserve, loss of ventilatory function, worsening of clinical symptoms, respiratory failure, and secondary damage. But actually we don’t know the complete long term consequences of hypercapnia [9]. Tsuboi [10] reported that persistent carbon dioxide retention in chronic ventilatory deficient subjects may reflect an adaptive mechanism that allows for lower levels of alveolar ventilation so as not to overload the respiratory muscles. In summary, carbon dioxide retention is involved in the respiratory center drive capacity, respiratory muscle strength, airway obstruction, pulmonary parenchymal damage, and many other complex processes. The gold standard for carbon dioxide retention is arterial blood gas analysis, but arterial blood gas analysis only reflects the instantaneous partial pressure of carbon dioxide in the blood. Therefore, checking blood gas at different times will produce different results, and the overall extent of COPD disease leading to carbon dioxide retention (or even respiratory failure) cannot be determined accurately. Accurate prediction of COPD carbon dioxide retention from pathological changes level would be of great help in disease monitoring.

What is the relationship between carbon dioxide retention and pulmonary function and imaging parameters? To date, this relationship has not been clear. For this study, we were interested in the ability to predict and estimate carbon dioxide retention using pulmonary function parameters and imaging parameters. Therefore, the aims of the present study were: 1) to compare the differences in clinical symptom scores, inflammatory markers, pulmonary function indicators, and CT parameters between patients with carbon dioxide retention in COPD vs those without carbon dioxide retention; and 2) to identify the factors associated with carbon dioxide retention in AECOPD.

## Methods

### Study design and subjects

This study was a retrospective study of consecutive AECOPD patients admitted to the Department of Respiratory Medicine of The Ninth Hospital of Xi’an Affiliated Hospital of Xi’an Jiaotong University from October 2014 to September 2017, meeting the GOLD diagnostic criteria (FEV_1_ / FVC < 70% bronchodilators inhaled). AECOPD refers to patients who have COPD symptoms (cough, sputum, shortness of breath, etc.) exacerbating the need for hospitalization. These patients are typically treated with a short acting bronchodilator, antibiotics, and / or a glucocorticoid. Exclusion criteria were as follows: 1) < 40 years of age; 2) pregnant women; 3) lung diseases such as lung cancer, pneumonia, active tuberculosis, pulmonary embolism, or interstitial lung disease; 4) previous pulmonary surgery; 5) unable to complete the pulmonary function test; 6) asthma, severe heart, liver, or kidney dysfunction; 7) CT images of insufficient quality for analysis; 8) other causes of respiratory failure such as obstructive sleep apnea syndrome; 9) Inclusion of AECOPD patients did not use NIV before blood gas analysis; and 10) prehospital treatment that included glucocorticoids or antibiotics.

This is a subgroup of the “Digital Lung” disease assessment system and diagnostic criteria (201402013) approved by the Chinese Society for Clinical Research (Grant No.: ChiCTR-OCH-14004904). The study was approved by the Ninth Hospital of Xi’an ethics committee (No.2014001). Written informed consent was obtained from all patients.

### Grouping

Among the included patients with AECOPD, those with arterial carbon dioxide partial pressure greater than 45 mmHg were assigned to the carbon dioxide retention group. Patients with arterial carbon dioxide partial pressure less than 45 mmHg were assigned to the non-carbon dioxide retention group (control group). The 45 mmHg was chosen as the threshold, rather than the diagnostic threshold of 50 mmHg for type II respiratory failure, because our interest was to study the differences in the characteristics of people with carbon dioxide retention and those without carbon dioxide retention. Of course, for treatment, this threshold is low, but for the study of carbon dioxide retention, we believe that the key point of ventilatory decompensation is more suitable, that is, arterial blood gas carbon dioxide partial pressure 45 mmHg. In addition, according to AECOPD treatment recommendations [11], PaCO2 ≥ 45 mmHg can also be used as a threshold for non-invasive ventilation treatment.

### Clinic and biochemistry data collection

A questionnaire was used to collect data on the participants’ sex, age, smoking status, body mass index (BMI), number of hospitalizations caused by AECOPD during the previous 12 months, the COPD Assessment Test (CAT), and the modified Medical Research Council (mMRC) dyspnea index at admission. Blood gas analysis was performed within 1 day of admission using a RADIOMETER ABL automatic blood gas analyzer (ABL800, RADIOMETER, Copenhagen, Denmark).

### Pulmonary function test (PFT)

Spirometry and impulse oscillometry (IOS) (MasterScreen, JAEGER, Germany) were performed before discharge. The maximum expiratory flow-volume curve, forced vital capacity, pulmonary diffusion function in one breath, and bronchial diastolic function were evaluated after administration of 200 μg of salbutamol (GlaxoSmithKline Pharmaceuticals Ltd.). The procedure was performed according to the ATS/ESR guidelines [12].

### 64-detector CT examination

Imaging examinations were performed using a 64-detector CT scanner (SOMATOM Definition AS, Siemens, Erlangen, Germany) with subjects holding their breath at full inspiration in the supine position. Technical parameters were based on our prior study [13] .

All CT images were automatically analyzed using the FACT-Digital Lung software [14, 15]. The percentage of the wall area (%WA) of different generations of bronchi in each lobe, the extent of emphysema in the whole lung, right lung, left lung, and emphysema heterogeneity index (HI) were expressed according to our prior study [13, 15, 16].

### Statistical analysis

Statistical analysis was performed with SPSS 19.0 (IBM, Armonk, NY, USA). Two-sided *P*-values < 0.05 were deemed statistically significant. Continuous data were tested for normality using the Kolmogorov-Smirnov test. Data meeting the normal distribution were expressed as mean ± standard deviation and were analyzed using Student’s test. Non-normally distributed data were expressed as median (range of 25th to 75th) and analyzed using the Mann-Whitney U test. Binary logistic regression models were used to identify predictive factors for the carbon dioxide retention group using a backward stepwise method, with a probability value for entry of *P* = 0.10 and removal of *P* = 0.05.

## Results

### Comparison of blood gas analysis data and other clinical parameters between the groups

Blood gas analysis showed that PaCO_2_ in the carbon dioxide retention group was 49.5 (46–57.75) mmHg, higher than 38 (35–41) mmHg in the non-retention group (*P* < 0.001). The pH in the carbon dioxide retention group was 7.39 (7.36–7.40), lower than 7.43 (7.41–7.45) in the non-retention group (*P* < 0.001), while PaO_2_ was 69 (58–89) mmHg in the carbon dioxide retention group, lower than 76.5 (67–85.5) mmHg in the non-retention group (*P* = 0.018).

Compared with the non-carbon dioxide retention group, the number of hospitalizations for the carbon dioxide retention group increased significantly during the 12 months prior to the study (*P* = 0.013); mMRC also increased (*P* = 0.034); There was no significant statistical difference between the groups for age, smoking, number of comorbidities, body mass index, CAT score (*P* > 0.05) (Table 1).

**Table 1:** Demographic and clinical datas between the carbon dioxide retention and non-carbon dioxide retention COPD

Varias	Carbon dioxide retention	Non-carbon dioxide retention	*p value*
	*n* = 86	*n* = 144	
Age, years	65.3 ± 9.43	67.53 ± 10.24	0.102
pack years	46.55 ± 32.49	41.62 ± 26.96	0.318
Number of hospitalizations in the past 12 months	0 (0–1.25)	0 (0–1)	0.013*
Comorbidities	1 (0–1)	1 (0–2)	0.369
BMI, kg/m^2^	22.97 ± 3.61	23.40 ± 3.80	0.449
CAT	21 (14–25)	18.5 (12.5–25)	0.18
mMRC	2 (1–3)	1 (0–2)	0.034*
WBC,*109/L	6.55 (5.24–8.07)	7.15 (5.5–9.12)	0.088
N,%	72.8 (63.7–80.5)	73 (62.75–81.2)	0.972
E,%	1.2 (0.5–2.4)	1.6 (0.6–3.2)	0.241
HB, g/L	144 (135–152)	142 (129.5–151)	0.142
PLT*109/L	155.5 (125–202)	169 (135–218)	0.057
FIB, g/L	3.64 (2.91–4.47)	4.06 (3.25–5.1)	0.02*
D-Dimer	0.89 (0.58–1.1)	0.87 (0.59–1.24)	0.843
CRP, mg/L	3.29 (3.28–13.15)	11.3 (3.28–36.3)	0.001**
PCT	0.05 (0.05–0.05)	0.05 (0.05–0.05)	0.203
PH	7.39 (7.36–7.40)	7.43 (7.41–7.45)	< 0.001***
PaO2, mmHg	69 (58–89)	76.5 (67–85.5)	0.018*
PaCO2, mmHg	49.5(46–57.75)	38 (35–41)	< 0.001***

*Abbreviations*: *BMI* body mass index, *GOLD* Global Initiative for Chronic Obstructive Lung Disease, *COPD* chronic obstructive pulmonary disease, *WBC* white blood cell count, *N* neutrophil, *E* Eosinophils, *HB* Hemoglobin, *PLT* blood platelet count, *FIB* fibrinogen, *CRP* C-reactive protein, *PCT* Procalcitonin

Note:*p < 0.05; **p < 0.01; ***p < 0.001

### Comparison of traditional lung function and IOS parameters between the groups

In traditional lung function tests, compared with the non-carbon dioxide retention group, the carbon dioxide retention group had lower FEV_1_, FEV_1_%pred, FEV_1_ / FVC, and MMEF_25–75%_ (*P* < 0.001), and higher RV/TLC (*P* = 0.017).

In the IOS test, compared with the non-carbon dioxide retention group, the carbon dioxide retention group possessed higher total airway resistance Z_5%pred_ and R_5%pred_ (*P* = 0.004 and 0.008, respectively), and higher peripheral airway resistance parameters R_5_-R_20_ and Ax (*P* = 0.009 and *P* = 0.011, respectively). X_5_ negative increase was more pronounced in the carbon dioxide retention group than the non-retention group (*P* = 0.022; Table 2).

**Table 2:** Comparison of traditional pulmonary function tests and pulsed oscillatory resistance determination between the carbon dioxide retention and non-carbon dioxide retention COPD

	Carbon dioxide retention (*n* = 86)	Non-carbon dioxide retention (*n* = 144)	*p* value
Z5%pred	185.9 (154–216.65)	162.4 (131.35–199.6)	0.004
R5%pred	168 (138.85–197.7)	148 (126.75–182.75)	0.008
R5	0.52 (0.43–0.61)	0.49 (0.39–0.57)	0.057
R20%pred	123.4 (105.75–139.55)	117.2 (104.3–135.6)	0.236
R20	0.32 (0.29–0.375)	0.33 (0.29–0.37)	0.764
R5-R20	0.18 (0.125–0.23)	0.14 (0.09–0.205)	0.009
X5	−0.24 (−0.31--0.16)	−0.19 (− 0.32--0.13)	0.022
Fres	22 (18.26–26.19)	21.2 (17.48–24.61)	0.173
Ax	1.89 (1.27–2.57)	1.54 (0.74–2.44)	0.011
FEV1	0.95 (0.77–1.42)	1.39 (1.06–1.74)	< 0.001
FEV1%pred	34.25 (25.4–47.8)	51.45 (40–61.7)	< 0.001
FEV1/FVC	48.14 (42.08–56.71)	56.26 (46.55–62.79)	< 0.001
MMEF75–25%pred	14.25 (9.7–19.9)	21.5 (16.1–27.75)	< 0.001
DLCO/VA	75.47 ± 24.29	80.13 ± 24.34	0.169
RV/TLC	59.44 ± 10.11	55.98 ± 10.63	0.017

*Abbreviations*: *PFT* Pulmonary function test, *FEV1* forced expiratory volume in 1 sec, *FVC* forced vital capacity, *FEV1/FVC* forced expiratory volume in 1 sec/forced vital capacity, *MMEF*_*25–75%*_ maximal mid expiratory flow, *RV/TLC* residual volume/total lung capacity, *DLCO/VA* ratio of carbon monoxide diffusion capacity to alveolar ventilation, *%Pred*, of the predicted value, *Z*_*5*_ Total respiratory impedance, *R5* resistance at 5 Hz, *R*_*20*_ resistance at 20 Hz, *X*_*5*_ reactance at 5 Hz, *Fres* response frequency, *Ax* reactance area

The above results show that the airflow restriction in the retention group was more obvious; total airway resistance and peripheral airway resistance were higher.

### Comparison of CT parameters between the groups

**Fig. 1:**
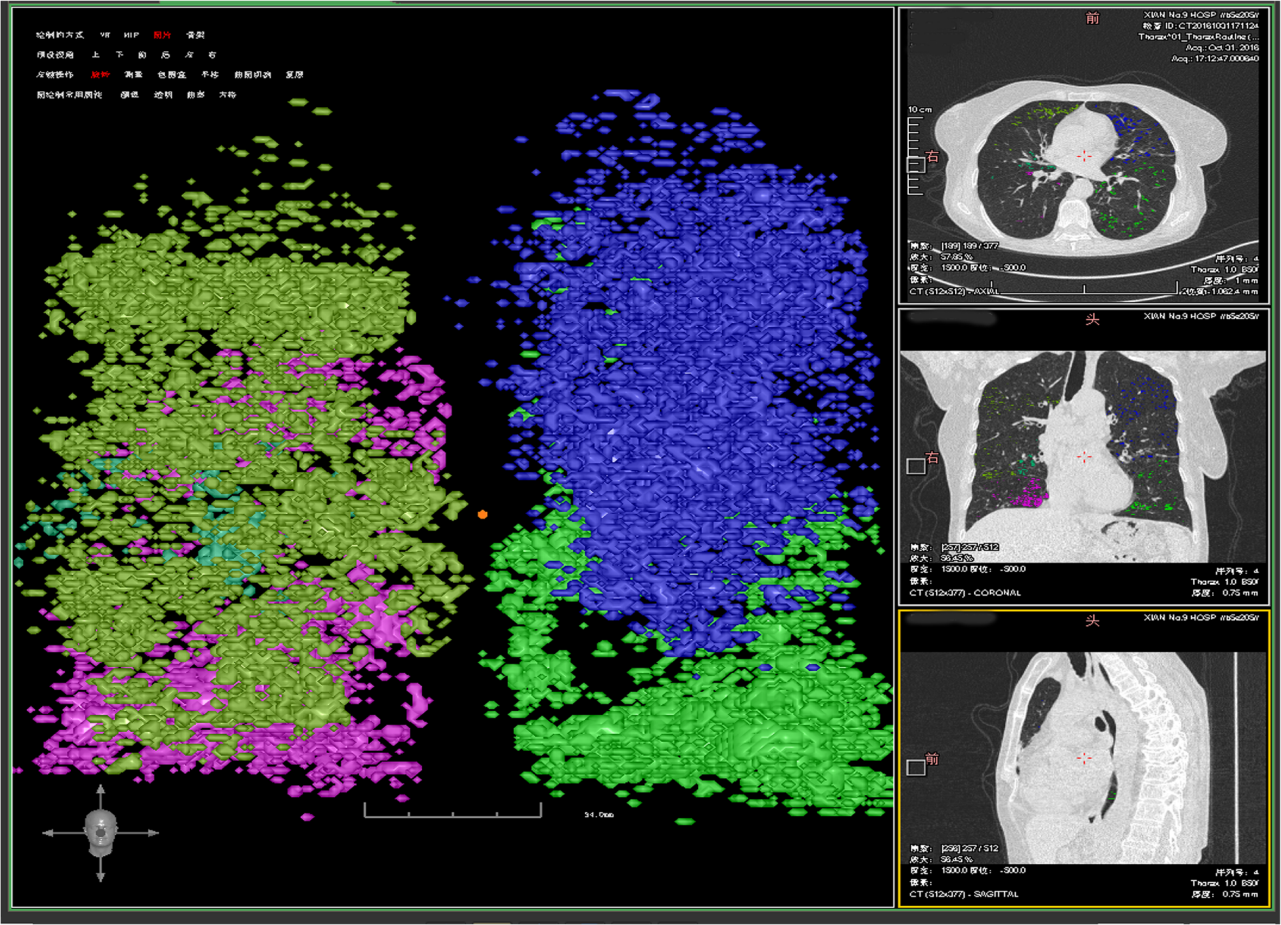
Lung volume, emphysema index and mean lung density in AECOPD patient without carbon dioxide retention (Volume_whole_4012.33 ml, EI_whole_9.82%, MLD_whole_-808.26Hu)

**Fig. 2:**
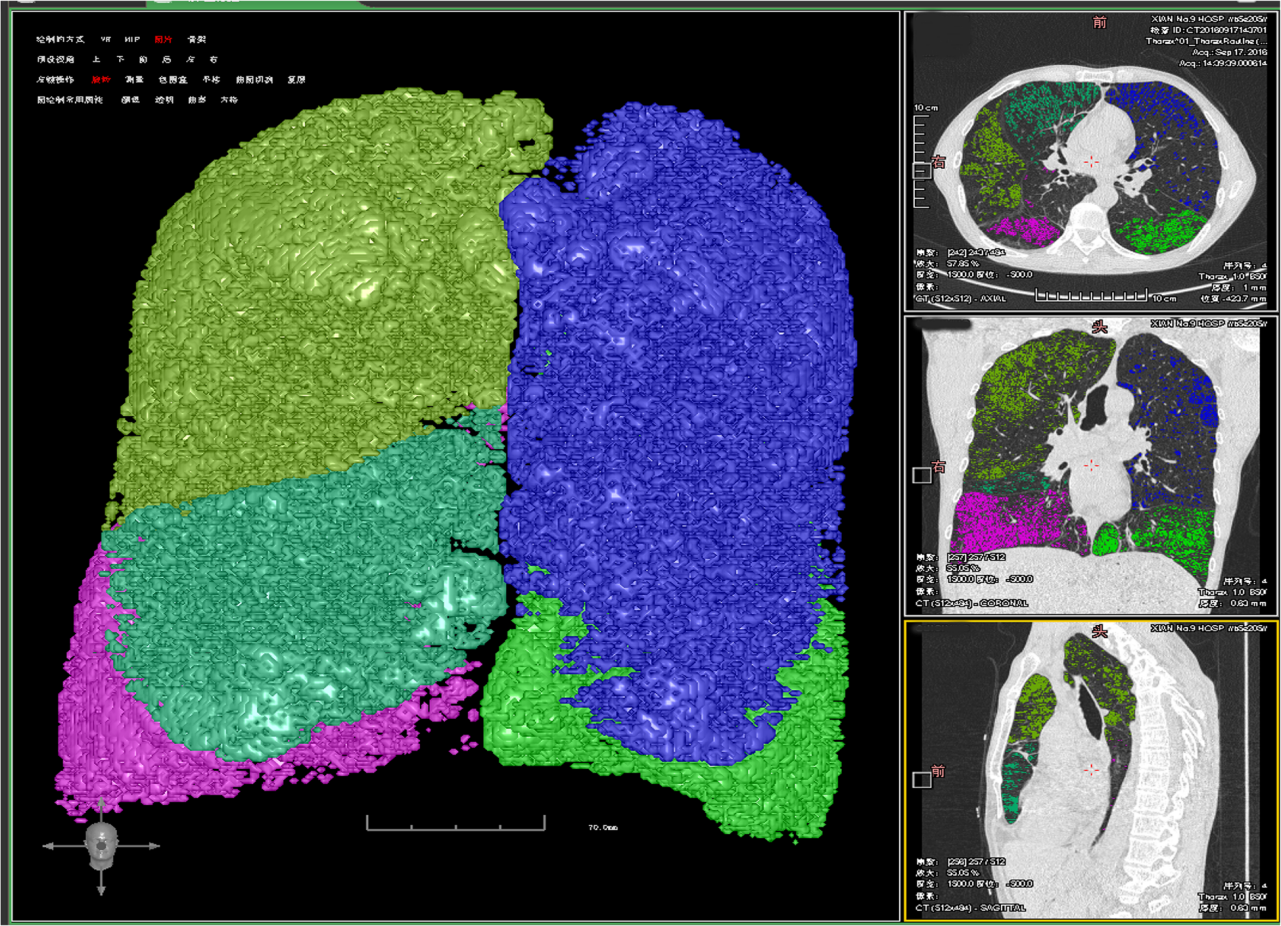
Lung volume, emphysema index and mean lung density in AECOPD patient with carbon dioxide retention (Volume_whole_6187.62 ml, EI_whole_15.17%, MLD_whole_-851.18Hu)

There was a statistical difference in total lung capacity, emphysematous index, and mean lung density between the groups. Compared to the non-carbon dioxide retention group, the carbon dioxide retention group had increased total lung volume [6106.56 (5113.8–6767.43) vs 5578.61 (4512.44–6459.67), *P* = 0.011], increased %LAA_whole_ [23.23 (15.43–29.51) vs 18.02 (11.83–25.83), *P* = 0.007], and lower mean lung density [− 861.37 (− 878.99--834.07) vs − 851.21 (− 867.76--829.83), *P* = 0.043] (Table 3). The above results show that the carbon dioxide retention group had more obvious increased lung volume and emphysema, and that pulmonary parenchyma damage was more pronounced (Figs. 1 and 2).

**Table 3:** Comparison of Emphysema variables between the carbon dioxide retention and non-carbon dioxide retention COPD

Emphysema variables	Carbon dioxide retention	Non-carbon dioxide retention	*p value*
Volume _whole_	6106.56 (5113.8–6767.43)	5578.61 (4512.44–6459.67)	0.011
%LAA _whole_	23.23 (15.43–29.51)	18.02 (11.83–25.83)	0.007
Mean lung density _whole_	−861.37(−878.99--834.07)	−851.21 (−867.76--829.83)	0.043
Volume _Right lung_	3180.79 (2756.52–3578.41)	3014.42 (2482.47–3488.95)	0.047
%LAA _Right lung_	23.4 (15.24–30.08)	17.65 (12.28–25.68)	0.006
Mean lung density _Right_	−859.2 (− 879.56--835.39)	−851.14 (− 866.61--829.63)	0.032
Volume _Left lung_	2878.78 (2319.57–3221.92)	2599.85 (2039.45–3039.15)	0.003
%LAA _Left lung_	23.39 (14.8–29.1)	18.83 (11.65–26.32)	0.008
Mean lung density _Left_	− 862.98 (−876.01--837.3)	− 847.85 (− 870.33--826.79)	0.039
HI _whole_	0.09 (−0.08–0.29)	0.16 (−0.02–0.32)	0.169
HI _Right lung_	0.15 (−0.06–0.35)	0.18 (− 0.02–0.38)	0.458
HI _Right lung_	− 0.17 (− 0.41–0.05)	−0.08 (− 0.28–0.11)	0.079

*Abbreviations*: *%LAA* the extent of emphysema of CT attenuation value below −950 HU;MDE:Mean density of emphysema;HI:emphysema heterogeneity index, when emphysemais equally distributed among the lobes or the full extent in the whole lung is < 1%, HI is near zero; otherwise,HI = (%LAAupper -%LAAlower)/(%LAAupper+%LAAlower)*100

There were no statistical differences in %WA_RUL4–7_, %WA_RML4–7_, %WA_RLL4–9_, %WA_LUL4–7_, and %WA_LLL4–9_ between the groups (*P* > 0.05; Additional file 1: Table S1).

#### Factors influencing carbon dioxide retention

Based on the results of the univariate analysis and their clinical significance, 6 parameters were entered into the logistics analysis: the number of hospitalizations because of AECOPD in the previous 12 months (≥1), mMRC (≥2), neutrophil ratio (≥70%), X5 negative increase (< 0), whole lung emphysema index (EI > 20%) and FEV_1_ (< 1 L). Logistic regression analysis, using the back stepwise method, showed that FEV_1_ < 1 L and %LAA_-950HU_ > 20% were independent risk factors for carbon dioxide retention (Table 4). The predictive value of these two parameters for carbon dioxide retention was 69.4%.

**Table 4:** Logistic regression analysis of factors associated with carbon dioxide retention

	*P*	OR (95% CI)
FEV1 < 1 L	< 0.001	4.011 (2.216–7.262)
%LAA-950Hu > 20%	0.026	1.926 (1.080–3.432)

## Discussion

COPD is a heterogeneous disease, and for the specific population included in this study, carbon dioxide retention indicates that the disease has progressed to the decompensation phase of respiratory dysfunction. In terms of COPD treatment, this population therefore requires the most medical resources and tends to respond poorly to clinical treatment. Our study revealed the clinical, pulmonary function, and imaging features of patients who have carbon dioxide retention. The carbon dioxide retention group had more frequent hospital admissions for acute exacerbations in the 12 months prior to the study, more pronounced dyspnea symptoms, and lower arterial partial pressure of oxygen. Regardless of the traditional lung function or IOS test, the carbon dioxide retention group had poorer parameters, more obstructive airflow, and higher residual volume. In imaging, the carbon dioxide retention group had higher lung volume and emphysema index, and lower mean lung density. However, there was no difference in emphysematous distribution and multi-stage bronchial wall area. Our results showed that FEV_1_ < 1 L and EI > 20% can help predict the increased risk of COPD with carbon dioxide retention.

Studies from ECLIPSE suggest that the clinical manifestations of COPD vary widely, and the extent of airflow limitation cannot capture the heterogeneity of the disease [17]. FEV_1_ is not believed to reflect the whole picture of COPD and is not a reliable predictor of disease stage for specific individuals [18]. However, there is also the opinion [19] that FEV_1_ < 1 L is an independent prognostic factor. Although FEV1 is generally expressed more accurately as a percentage of the predicted value, a fixed cutoff is assessed for limited airflow severity when FEV1 is much lower than normal, and we believe it to be reliable for clinical use. Our study also showed that FEV_1_ < 1 L predicts the presence of carbon dioxide, which is especially useful for assessing COPD with chronic long-term carbon dioxide retention and to provide a reference for deciding on treatments like adjuvant ventilation, or for parameter selection as a follow-up step. Emphysema index is currently a more accepted imaging parameter for the assessment of COPD [20, 21] because of its reflection of both pathological and functional impairments. Our regression analysis showed that EI > 20% and FEV_1_ < 1 L can be used to predict carbon dioxide retention, reflecting both pathological and functional impairments. O’Donnell [22] conducted a similar study using discriminant analysis and found that FEV_1_ / FVC rates, as well as vital capacity (% predicted) or FVC (% predicted), differentiated patients requiring mechanical ventilation from those who did not.

There are many phenotypes based on COPD [23], however, COPD with carbon dioxide retention has rarely been studied. Gas exchange in COPD is very complicated; the mechanism of carbon dioxide retention induced hypercapnia is the result of multiple pathological processes that are interwoven at varying degrees and affected by the disease process itself. In addition, the cellular and molecular details of lung tissue destruction are not completely understood [24]. The destruction of lung parenchyma mainly manifested as emphysema, accompanied by pulmonary vascular bed damage. Small airway remodeling and occlusion are other important outcomes of pathological damage. In acute exacerbation events, airway spasms, mucosal edema, and sputum cause increased airway obstruction and inflammation.

Chronic respiratory failure results in carbon dioxide retention due to respiratory insufficiency [25, 26], We found that there was a more severe airflow limitation in the carbon dioxide retention group. In the image data for this group, we found a more obvious increase in the lung volume and the emphysema index, and that the mean lung density was lower, suggesting that there was not only excessive expansion of dynamic lung, but also more physical damage to the lung involved in the pathological process.

Clinical strategies for AECOPD include: treatment of the primary disease, controlled oxygen therapy, and the use of an invasive or non-invasive ventilator to improve lung ventilation. Current clinical treatment is partial to improving lung ventilation, while putting less emphasis on changes in the pulmonary parenchyma. However, better carbon dioxide removal has been a topic of growing interest in recent years, and a new approach involves extracorporeal venous CO_2_ removal [27–29].

Carbon dioxide retention in the body can cause harm that is multi-system and widespread. Clinical emphasis is on the treatment of hypoxia, but there is an attitude of tolerance to carbon dioxide retention. Clinicians should recognize that carbon dioxide retention will increase the hypoxic damage to multiple tissues [30, 31]. Hypervolemic respiratory failure noninvasive ventilation (NIV) treatment is the primary method of clinical management and, based several large studies [32, 33], it is reasonable to use a higher level of partial pressure of carbon dioxide to determine NIV use. Our study focused on the parameters of pulmonary function and imaging that would be valuable when carbon dioxide retention was elevated, so the cutoff partial pressure of carbon dioxide was chosen as 45 mmHg.

The characteristics of COPD populations with carbon dioxide retention are typically not of concern. Most studies focus on the COPD populations based on pulmonary function grading. In contrast, our research is novel because we focused on a particular population with specific clinical features, and so were able to obtain some valuable results. However, the present study is not without limitations. It is only assumed that persistent carbon dioxide retention and transient carbon dioxide retention are different, but there are no observations for the longitudinal outcomes of these conditions. Secondly, the sample size was small. To address these issues, more research is needed to explore the features of carbon dioxide retention in patients with AECOPD.

## Conclusion

The carbon dioxide retention COPD group had more airflow obstruction and higher residual volume, lung volume, and emphysema index, as well as lower mean lung density compared to the COPD group without carbon dioxide retention. FEV1 < 1 L and EI > 20% may be predictors of an increased risk of carbon dioxide retention.

## Additional file

Additional file 1: **Table S1.** Comparison of wall areas between the carbon dioxide retention and non-carbon dioxide retention COPD groups. (XLSX 10 kb)

## Abbreviations

%Pred: of the predicted value
%WA: the percentage of the wall area; the %WA of different generations in each lobes were represented as %WARUL4–7, %WARML4–7, %WARLL4–9, %WALUL4–7, and %WALLL4–9%LAA the extent of emphysema of CT attenuation value below − 950 HU
Ax: reactance area
BMI: body mass index
COPD: chronic obstructive pulmonary disease
CT: computed tomography
DLCO/VA: ratio of carbon monoxide diffusion capacity to alveolar ventilation
E: eosinophil
FEV1: forced expiratory volume in one second
FEV1/FVC: forced expiratory volume in one second/forced vital capacity
Fres: resonant frequency
FVC: forced vital capacity
GOLD: Global Initiative for Chronic Obstructive Lung Disease
HB: Hemoglobin
HI: emphysema heterogeneity index
MED: mean emphysema density
MMEF25–75%: maximal mid expiratory flow
mMRC: modified Medical Research Council
N: neutrophil
NIV: noninvasive ventilation
PFT: Pulmonary function test
PLT: blood platelet count
R20: resistance at 20 Hz
R5: resistance at 5 Hz
RV/TLC: residual volume/total lung capacity
WBC: white blood cell count
X5: reactance at 5 Hz
Z5: Total respiratory impedance
